# Geographical disparities and determinants of childhood diarrheal illness in Ethiopia: further analysis of 2016 Ethiopian Demographic and Health Survey

**DOI:** 10.1186/s41182-020-00252-5

**Published:** 2020-08-03

**Authors:** Asmamaw Atnafu, Malede Mequanent Sisay, Getu Debalkie Demissie, Zemenu Tadesse Tessema

**Affiliations:** 1 Department of Health Systems & Policy, Institute of Public Health, College of Medicine and Health Sciences, University of Gondar, Gondar, Ethiopia; 2 Department of Epidemiology and Biostatistics, Institute of Public Health, College of Medicine and Health Sciences, University of Gondar, Gondar, Ethiopia; 3 Department of Health Education and Behavioral Science, Institute of Public Health, University of Gondar, Gondar, Ethiopia

**Keywords:** Spatial statistics, Ethiopia, Under-five children, Diarrhea, Generalized mixed model

## Abstract

**Background:**

Childhood diarrheal illness is the second leading cause of child mortality in sub-Saharan Africa, including Ethiopia. Epidemiology of diarrhea has long-term implications with respect to medical, social, and economic consequences. Studies hypothesize that there have been regional differences, and this study aimed to examine the spatial variations and identify the determinants of childhood diarrhea in Ethiopia.

**Methods:**

Data from the 2016 Demographic and Health Survey of Ethiopia (EDHS), which included 10,337 aged under 5 years were analyzed. The survey was conducted using a two-stage stratified sampling design. The study attempted to detect and test the clustering of diarrhea cases using global Moran’s I and LISA. Descriptive statistics followed by mixed-effect logistic regressions were used to identify factors related to the prevalence of diarrhea.

**Results:**

Overall, 11.87% of the children experienced childhood diarrheal illness. The study showed that the risk was high in the southern and central parts and low in the eastern and western regions of the country. Children aged 6–12 (AOR = 2.66, [95% CI 2.01, 3.52]), 12–23 (AOR = 2.45, [95% CI 1.89, 3.17]), and 24–35 (AOR = 1.53, [95% CI 1.17, 2.01]) months were more likely to suffer from childhood diarrhea than those aged less than 6 months. Children in Tigray (AOR = 1.69 [95% CI 1.01, 2.83]), Amhara (AOR = 1.80, [95% CI 1.06, 3.06]), SNNPR (AOR = 2.04, [95% CI 1.22, 3.42]), and Gambella (AOR = 2.05, [95% CI 1.22, 3.42]) were at higher risk than those in Addis Ababa. The odds of getting diarrhea decreased by 24% among households with ≥ 3 under-five children compared to those with only one under-five child (AOR = 0.76 [95% CI 0.61, 0.94]). The odds of getting diarrheal illness for the children of employed mothers increased by 19% compared to those children of non-employed mothers (AOR = 1.19 [95% CI 1.03, 1.38]).

**Conclusions:**

Childhood diarrheal disease is prevalent among under-five children, particularly in the regions of SNNP, Gambella, Oromia, and Benishangul Gumuz, while the regions are generally making progress in reducing childhood illness. Capacity building programs with the best experience sharing and better home environments can be effective in reducing the incidence of childhood diarrhea in Ethiopia.

## Background

Although considerable progress has been made in reducing mortality from 93 to 39 deaths per 1000 live births between 1990 and 2018, this remains a major public health issue. Today, the differences between high- and low-income countries remain important in infant mortality [1]. Out of all causes, infectious diseases have been responsible for the greatest global burden of death and disability among children under 5 years of age [2–5]. Water-related diseases, such as diarrhea, malaria, and pneumonia, are the main causes of under-five mortality in the majority of low-income countries and are still prevalent, undermining the achievement of international obligations and reflecting social inequalities [6–11]. Only diarrhea is responsible for more than half a million deaths per year in low- and middle-income countries more than HIV/AIDS, malaria, and measles combined [12–16]. It is the leading cause of death among children under the age of 5 and kills approximately 525,000 children every year with most occurring in Africa countries [17–19]. For example, according to the Global Burden of Disease study in 2016, 9.4% of all severe cases of diarrhea in 2015 occurred in two countries, Ethiopia and the Democratic Republic of Congo. Also, diseases rise over 15 years have been seen in Central Africa, Gabon, Ivory Coast, Nigeria, and Zimbabwe [12, 20, 21].

Children’s death occurred mainly due to diarrhea, malaria, and pneumonia in Ethiopia. Diarrheal disease is the main cause of death and morbidity in children aged less than 5 years. It is the second leading cause of death in children under 5 years of age accounting for 30% of all annual deaths [22, 23]. Childhood diarrhea distribution is heterogeneous in various regions of Ethiopia, despite ongoing efforts to invest and improve through education and improving child health care via routine vaccination [24–26].

Many young children in Ethiopia die from pneumonia and diarrhea, which can easily be prevented by improving family and community health practices. Principally, it means ensuring the combined treatment of major childhood illnesses, emphasizes disease prevention through immunization, and strengthens nutrition, in particular, by raising awareness and promoting hygiene. As indicated by the national and regional facilities reports, diarrhea is one of the top five causes of morbidity and mortality among under-five children, which is considered to be the best overall indicator of children’s well-being [22]. Moreover, community-based studies revealed that the pooled prevalence of diarrhea among under-five children in Ethiopia was 22% [27–31]. A recent population-based survey also found that the prevalence of diarrhea was 12% [32]. *S*tudies have also shown unequal distributions of the burden of childhood diseases in different regions of the country [24, 33].

Similarly, diarrheal conditions are largely associated with livelihood, poverty, lack of hygiene, and water in households. The disparities between cities, villages, communities, and countries with clean water, sanitation, drainage, and waste disposal are factors that cause inequalities in diarrheal morbidity and death [34]. Moreover, existing empirical evidence emphasizes the prevalence of diarrhea and modeled an individual’s characteristics associated with the disease using standard logistic regression, which ignores clustering effects [35–41]. However, observations within a cluster tend to be more similar to observations, and ordinary analyses that ignore this may be inappropriate [42]. Ignoring clustering in analyses may overstate or understate the precision of results; risk factors may be incorrectly stated as significant [43]. Thus, having considered the limitations of existing studies, this analysis was conducted to identify factors that are associated with diarrhea using the generalized linear mixed model (GLMM), which considers the correlation between responses of interest to respondents from within the same cluster [44].

Spatial techniques also help to identify hotspots and provide information that enables public health officers and policymakers in strategic planning [45]. Local estimates of the diarrheal burden can be used to prioritize diarrheal care and prevention interventions among marginalized populations living in remote or conflict areas. In order to provide strength towards universal health coverage, as envisaged in the sectoral strategy, an enhanced understanding of who and where disadvantaged and vulnerable children are, is therefore critical. Throughout this end, recognizing differences in stunting is essential to the design of equity-focused interventions. To the best of our knowledge, there is a scarcity of published information on the risk factors of diarrhea with a triangulated spatial analysis of its prevalence in the country. To monitor the health status of the population and to evaluate the use and effectiveness of disease protection and control measures, up-to-date information is required. This study aimed to address the social determinants and burden of diarrheal diseases among children under 5 years of age in Ethiopia.

## Methods

### Study design, data sources, and population

The study utilized data from the nationally representative cross-sectional Ethiopian Demographic and Health Survey conducted in 2016 and included samples of households obtained through a two-stage stratified sampling procedure [32]. In the first stage, the country was divided into 21 strata and a total of 645 enumeration areas (EAs) ( considered as primary sampling units) were selected independently from each stratum using the probability proportional to the size technique. In the second stage, a systematic sampling technique was employed to select 30 households from each of the EAs. Finally, married women aged 12 to 49 years living in the selected households were approached for the interviews. The study used information from 10,641 children under the age of 5 years born to women living in households.

The morbidity data contained in the survey came from mothers’ responses to questions on recent episodes of various forms of morbidity. The mothers were asked if their children had a fever, cough, short rapid breaths, or diarrhea within the 2 weeks preceding the survey [32].

### Variables of study

In this study, we used the data for birth history information of all women aged 15 to 49 interviewed for different surveys. The birth history data set contained information on the date of birth of all children a woman had in her life starting from her first child until the time of the survey. Information on child survival (dead or alive) was also collected.

The outcome variable from EDHS 2015/16 used in this study was diarrhea episodes in children under 5 years of age during the 2 weeks before the interview. Diarrhea was measured using the definition of a child with loose stools more frequently than usual in the 2 weeks preceding the survey [32]. We constituted a binary variable denoting “one” if present and “zero” if absent.

The exposure variables included information regarding the socio-demographic and economic characteristics of children less than 5 years of age obtained from interviews with their mothers/caregivers. We selected explanatory variables based on prior studies, epidemiological information, review of published demographic studies, and information in the EDHS datasets. Location data (latitude and longitude coordinates) were also taken from selected enumeration areas. The survey datasets and location data were accessed through the web page of the International DHS program after subscription and authorized as a user. Of the total 643 clusters, 429 were considered for the spatial analysis of childhood diarrhea (21 dropped for lack of coordinate data set and 193 because they had no diarrhea cases).

### Data analysis

#### Spatial analysis

Spatial cluster detection was performed to identify cluster locations or the randomness of the observed pattern with a higher prevalence of diarrhea. A well-known method for describing dependencies between rates measured in spatial units is the spatial autocorrelation coefficient I first proposed by Moran. The global spatial autocorrelation (Moran’s I) was used to measure the overall clustering of the data and to project the strength and pattern of spatial autocorrelation [46]. Also, incremental autocorrelation was done to identify the distance band where spatial processes promoting clustering were most pronounced. The proportion of children suffering from diarrhea significant hot and cold spots of areas was identified by using LISA analysis (Local Moran I). Hotspots are geographical units with a high prevalence of diarrhea surrounded by other geographic units with similar prevalence. Similarly, a cold spot is a geographic location with low diarrhea prevalence surrounded by other geographic units with low diarrhea prevalence. High-high and low-low districts suggested the clustering of geographies with similar values of diarrhea prevalence, whereas high-low and low-high districts indicated spatial outliers. A *p* < 0.05 was considered significant throughout. The spatial interpolation technique was also used to predict childhood diarrhea in unsampled areas in the country based on sampled EAs. The Ordinary Kriging spatial interpolation method was used to predict childhood diarrhea in unobserved areas.

The SaTScan spatial statistics analysis was also used to identify purely spatial clusters of childhood diarrhea. Scan statistics were scanned gradually across the space to identify the number of observed and expected observations inside the window at each location. The scanning window with a maximum likelihood was the most likely high-performing cluster, and a *p* value was assigned to this cluster [47].

#### Regression analysis

In exploring the association between these variables and the incidence of childhood diseases, both bivariate and multivariate approaches were employed. Considering the design of the DHS survey, we accounted for the clustering of diarrhea by primary sampling units and included a random effect in the analysis. Thus, the mixed effect logistic regression was fitted: a model, which is the most appropriate for a correlated dependent variable. Model comparison was done based on Akaike information criteria (AIC), Bayesian information criteria (BIC), and intracluster correlation (ICC) values. The model with the lowest AIC was chosen. For analysis, the risk (adjusted odds ratio (AOR)) of diarrhea was assessed relating to socioeconomic variables. *p* values less than 0.05 were considered significant variables in the model. The analysis was performed using STATA, ArcGIS, and SaTScan.

## Results

### Socioeconomic characteristics

A total of 10,377 under-five children were included in the study. Table 1 highlights the distribution of participants across different characteristics. The majority of children 9187 (88.87%) were from rural areas. The mean age of the mothers was 29.56 (± 6.59) with 53% of whom were between the ages of 25 and 34 years. More than half of the mothers, 5744 (55.56%) and 6809 (65.87%) had no work and formal education, respectively. Only 55.42% had access to improved sources of water and only 10% had improved toilet facilities. Only 20% were exposed to the media (Table 1). Overall, 11.87% of the children in Ethiopia experienced childhood diarrheal illness in the 2 weeks before the survey.

**Table 1:** Socio-demographic characteristics of mothers, parents, and children under 5 years of age, DHS 2016, Ethiopia

Characteristics	Categories	Weighted frequency (*n* = 10,377)	Weighted %
Region	Tigray	682	6.60
	Afar	104	1.00
	Amhara	1954	18.90
	Oromia	4537	43.89
	Somali	473	4.57
	Benishangul	113	1.10
	SNNP	2149	20.79
	Gambella	25	0.24
	Harari	24	0.23
	Addis Ababa	233	0.26
	Dire Dawa	43	0.42
Mother’s education level	No education	6809	65.87
	Primary	2777	26.87
	Secondary/higher	751	7.27
Partner’s education status	No education	5280	51.08
	Primary	3849	37.24
	Secondary/higher	1208	11.69
Mother’s age in years	15–24	2288	22.14
	25–34	5501	53.22
	35–49	2548	24.65
Residence	Urban	1151	11.13
	Rural	9187	88.87
The current age of children (years)	< 6	1195	11.56
	6–11	1069	10.34
	12–23	2001	19.35
	24–35	1927	18.64
	36–47	1980	19.16
	48–49	2166	20.95
Number of children under 5 years	1	3901	37.74
	2	4640	44.74
	≥ 3	1796	17.37
Mother’s occupation	Not working	5744	55.56
	Working	4593	44.44
Wealth index	Poor	4848	46.89
	Middle	2139	20.69
	Rich	3350	32.41
Source of drinking water	Improved water	5728	55.42
	Unimproved water	4609	44.58
Toilet facilities type	Improved	1036	10.02
	Unimproved	9301	89.97
Child stool disposal	Safe	2797	27.06
	Unsafe	7540	72.94
Sex of child	Male	5307	51.34
	Female	5030	48.66
Marital status	Married	9715	93.98
	Not married	622	6.02
Duration of breastfeeding	Ever breastfed, not currently breastfeeding	5159	49.90
	Never breastfed	376	3.64
	Still breastfeeding	4802	46.46
Media exposure	Exposed	2101	20.33
	Not exposed	8236	79.67
Diarrhea status of under-five children	No	9110	88.87
	Yes	1227	11.87

### Distribution of childhood diarrhea

**Fig. 1:**
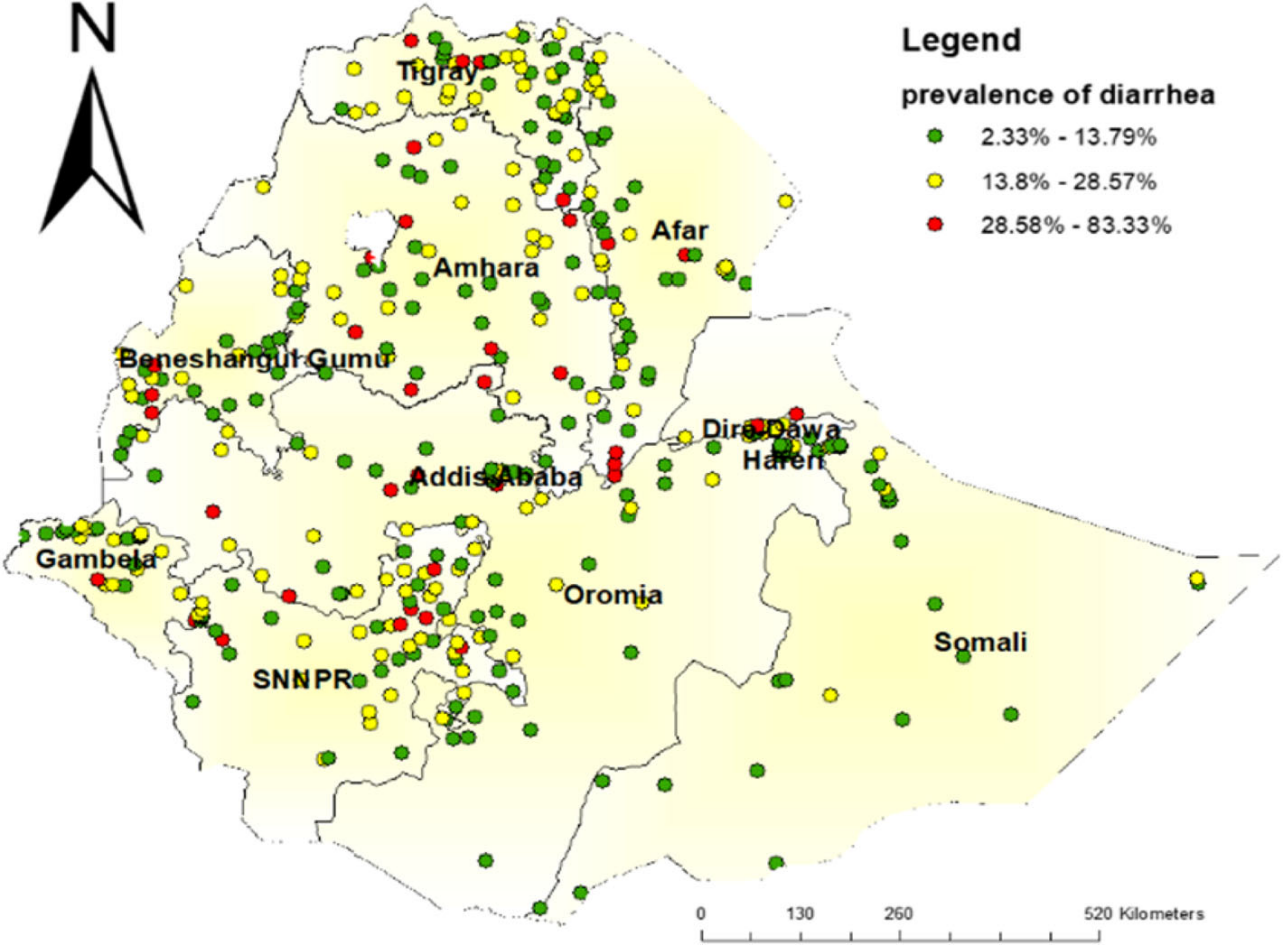
Spatial distribution of childhood diarrheal disease among under 5 years of age children in Ethiopia, EDHS 2016

Overall, the geographic distribution of childhood diarrhea at the cluster level is presented in Fig. 1. Generally, a high proportion of childhood diarrheal cases (red dots) and a low proportion of childhood diarrhea (green dots) were observed among under 5 years of age children in Ethiopia (Fig. 1).

### Spatial patterns of childhood diarrhea

**Fig. 2:**
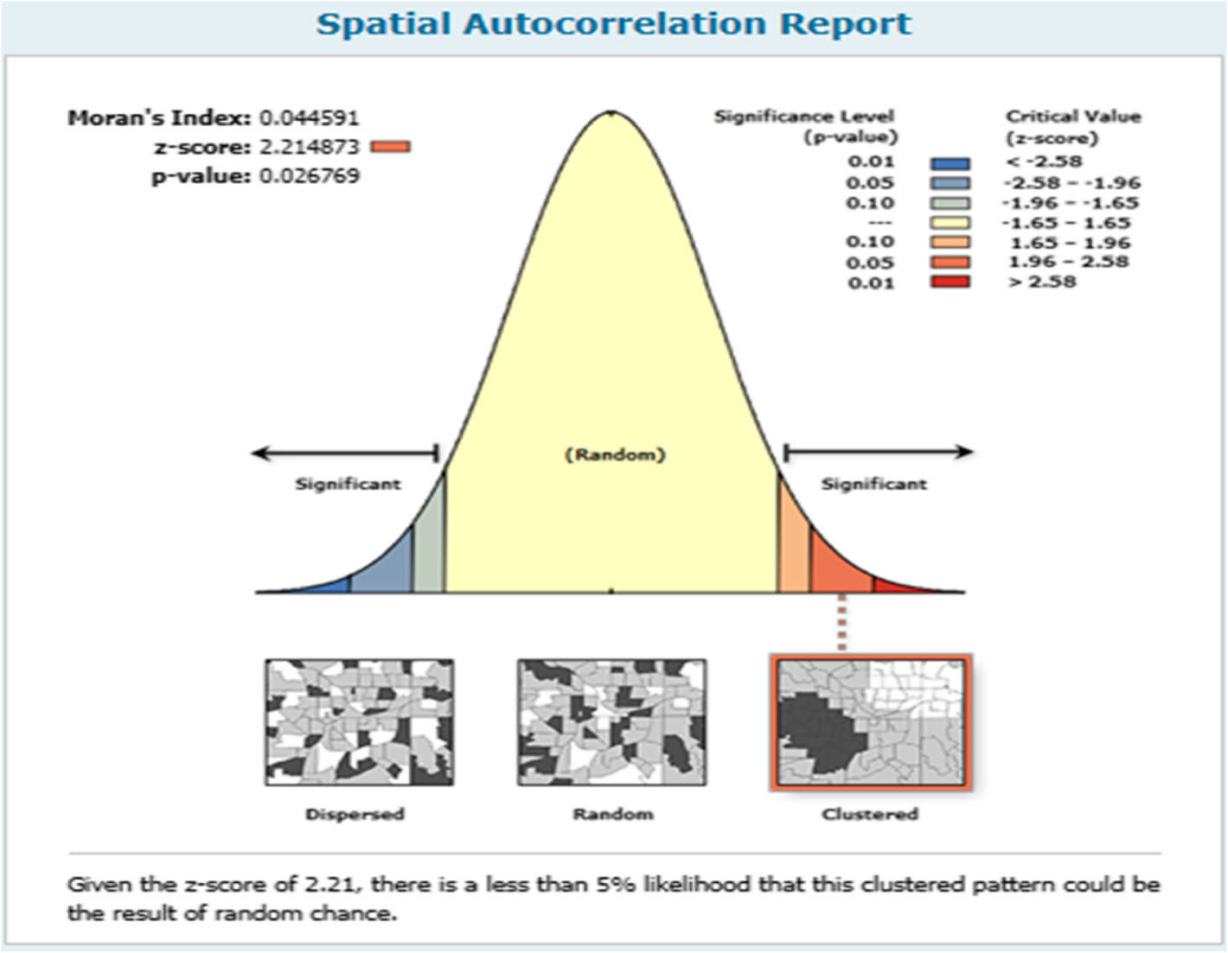
Spatial autocorrelation of childhood diarrheal disease among under-five children in Ethiopia, EDHS 2016

The spatial patterns of childhood diarrhea were found in the country. The global Moran’s I value (0.044591) indicated that there was significant clustering of childhood diarrhea in the country. There was a statistically significant spatial variability in childhood diarrhea among under-five children in Ethiopia (Fig. 2).

### Incremental spatial autocorrelation

**Fig. 3:**
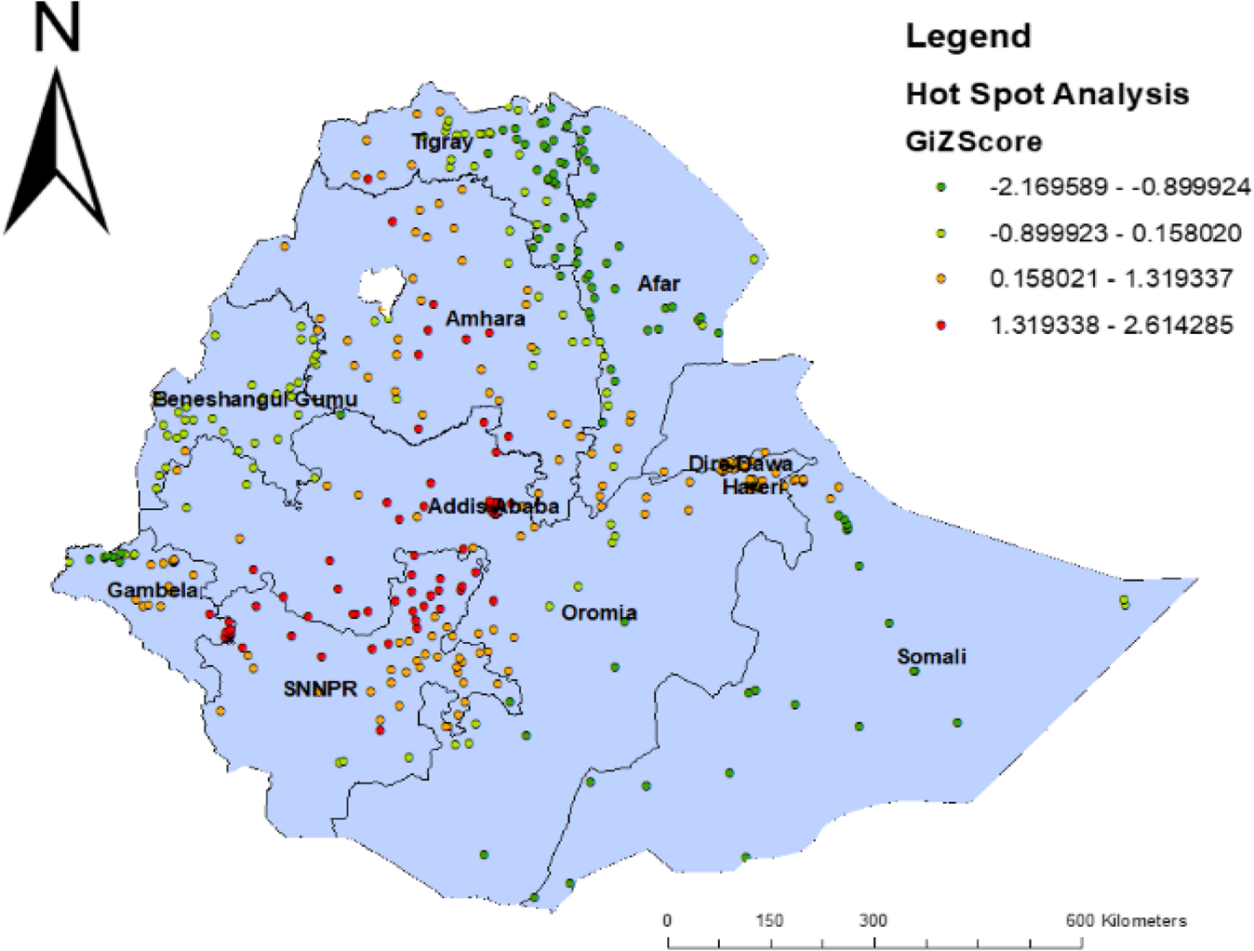
Hot spot analysis of childhood diarrheal disease among under 5 years of age in Ethiopia, EDHS 2016

Incremental spatial autocorrelation was made to determine the average closest neighbor, minimum, and maximum distance band for several distances presented in a line graph with the appropriate Z-score. A total of 10 distance bands were detected at an initial distance of 129,550 m, and the first peak (clustering) was observed at 167,402 m (Fig. 3).

### Hot spot analysis of childhood diarrhea

Figure 3 indicated the geographical distribution of childhood diarrhea. The hot spot regions were SNNP, Amhara, Addis Ababa, and Oromia regions, whereas the East Oromia, Benishangul Gumuz, Harari, Somali, Gambella, Afar, and North Tigray regions were indicated as cold spot areas.

### Spatial interpolation

**Fig. 4:**
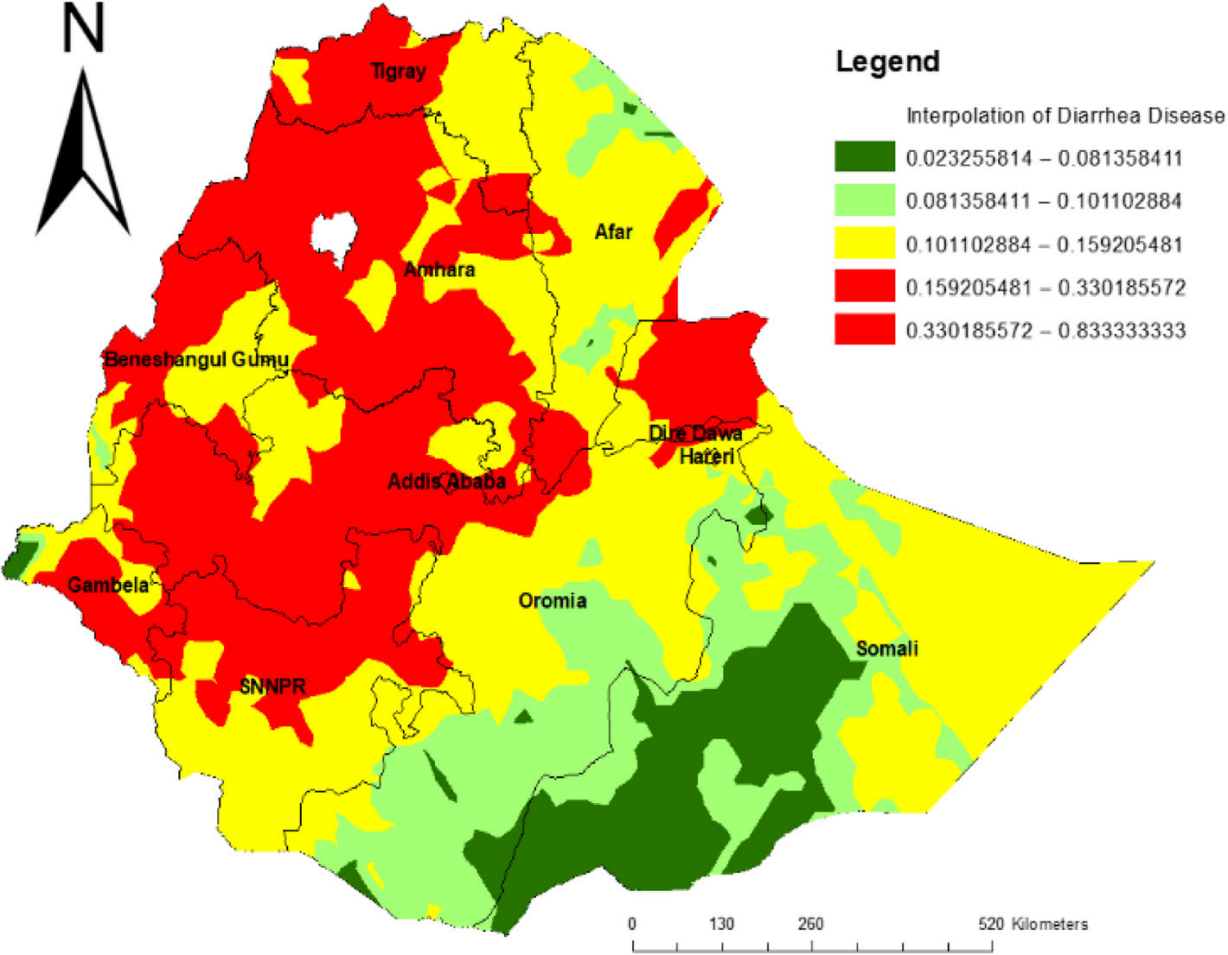
Spatial interpolation of childhood diarrhea disease among under-five age in Ethiopia, EDHS 2016

The red areas show predicted risk regions and children living in these areas were vulnerable to childhood diarrhea. In the first panel, western Tigray, Amhara, eastern Oromia, and northern SNNP regions were predicted as more risky areas compared to other regions. In the middle panel, Afar, southern Oromia, SNNP, and eastern Somali were identified as risk areas (Fig. 4).

### Spatial SaTScan analysis

**Fig. 5:**
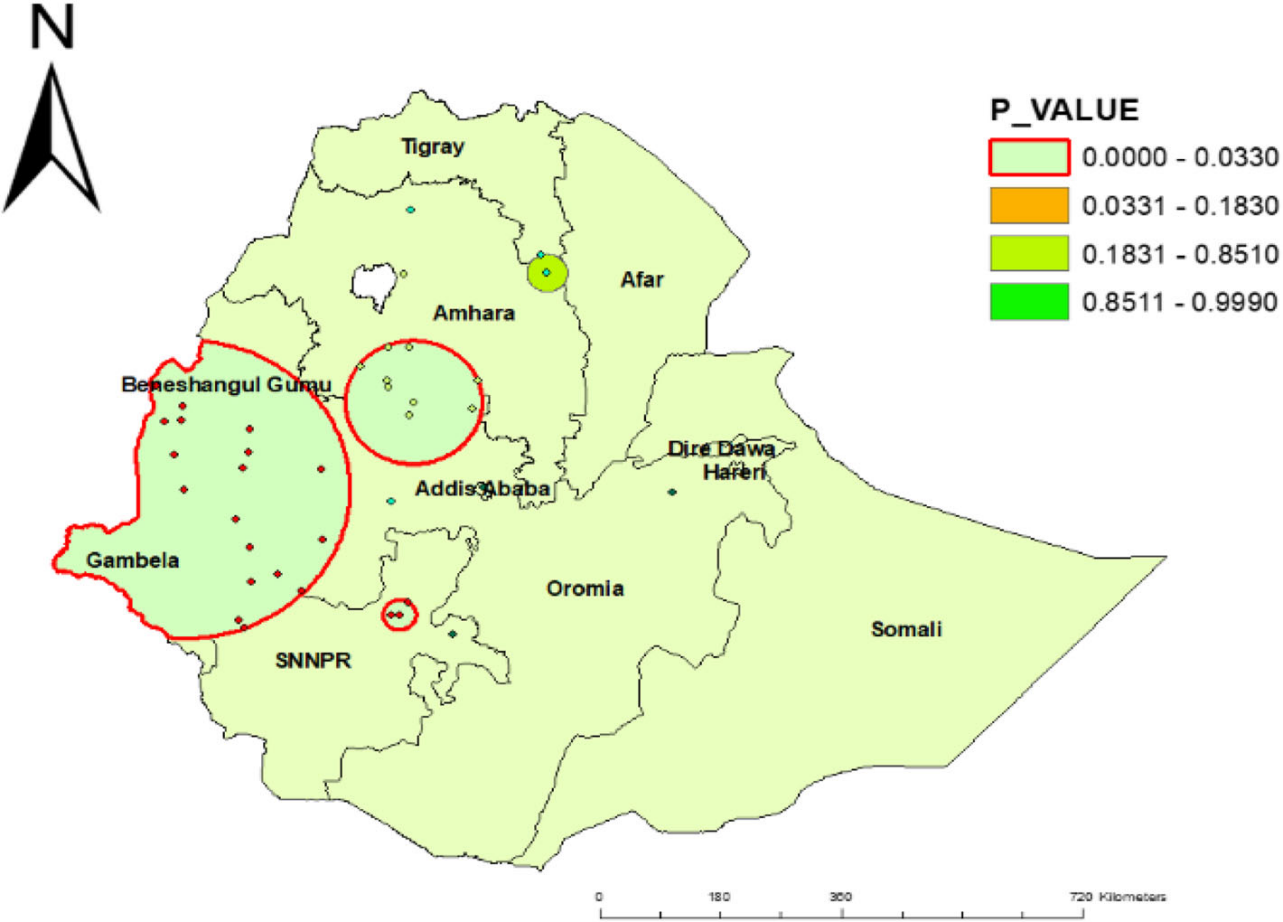
SaTScan results of childhood diarrheal disease among under-five children in Ethiopia, EDHS 2016

A total of 21 significant clusters were identified. Of these, 3 were most likely (primary) and 18 were secondary clusters. The spatial window of the primary cluster was located in the west SNNP, which was centered at (7.146476 N, 37.651928 E)/24.44 km, RR = 2.57, and log-likelihood ratio (LLR) of 17.84 at *p* < 0.001. It showed that children in the spatial window had 2.57 times higher childhood diarrhea than those outside the window. The spatial window of the secondary cluster was located in the Gambella, Oromia, and Benishangul Gumuz regions centered at (8.989285 N, 34.767792 E)/243.09 km, RR = 1.50, and log-likelihood 14.39 with *p* value < 0.001 (Table 2, Fig. 5).

**Table 2:** SaTScan analysis results of childhood diarrhea among under-five children in Ethiopia, 2016

Cluster	EA (enumeration area)	Coordinate or radi	RR	LLR	*p* value
Primary (3)	565, 126, 360	(7.146476 N, 37.651928 E)/24.44 km	2.57	17.84	< 0.001
Secondary (18)	248, 462, 558, 304, 433, 349, 165, 407, 555, 88, 285, 177, 586, 294,62, 437, 489, 325	(8.989285 N, 34.767792 E)/243.09 km	1.60	14.39	< 0.001

### Factors associated with childhood diarrhea

According to the multilevel multivariable logistic regression analysis, child age, a number of under-five children in a household, mother’s occupation, and region were statistically significant determinant factors of childhood diarrhea. Regarding regions, children in Tigray region (AOR = 1.69 [95% CI 1.01, 2.83]), Amhara (AOR = 1.80 [95% CI 1.06, 3.06]), SNNPR (AOR = 2.04 [95% CI 1.22, 3.42]), and Gambella (AOR = 2.05 [95% CI 1.22, 3.42]) had the highest odds of getting diarrhea compared to children in Addis Ababa.

The age of children in months was a significant factor affecting childhood diarrhea. The odds of developing diarrheal disease among children aged 6 to 12, 12 to 23, and 24 to 35 months were AOR = 2.66 [95% CI 2.01, 3.52], AOR = 2.45 [95% CI 1.89, 3.17], and AOR = 1.53 [95% CI 1.17, 2.01] times higher than children aged less than 6 months, respectively. The odds of getting diarrhea among children aged 48 to 59 months decreased by 51% compared to children aged less than 6 months (AOR = 0.49 [95% CI 0.35, 0.66]). The odds of getting diarrhea decreased by 24% among households with 3 or more under-five children compared to households with only one under-five child (AOR = 0.76 [95% CI 0.61, 0.94]). The odds of getting diarrhea among children born from working mothers increased by 19% compared to those of non-working mothers (AOR = 1.19 (95% CI 1.03, 1.38)) (Table 3).

**Table 3:** Multilevel multivariable logistic regression analysis of childhood diarrhea among under-five children in Ethiopia, 2016

Characteristics	Categories	Crude OR (95% CI)	Adjusted OR (95% CI)
Region	Addis Ababa	1	1
	Tigray	1.84 (1.16, 2.91)	1.69 (1.01, 2.83) *
	Afar	1.56 (0.97, 2.49)	1.66 (0.97, 2.85)
	Amhara	1.94 (1.23, 3.08)	1.80 (1.06, 3.06) *
	Oromia	1.57 (1.01, 2.47)	1.52 (0.90, 2.55)
	Somali	0.82 (0.51, 1.32)	0.91 (0.53, 1.55)
	Benishangul	1.24 (0.76, 2.03)	1.19 (0.68, 2.08)
	SNNP	2.08 (1.33, 3.26)	2.04 (1.22, 3.42) *
	Gambella	1.93 (1.19, 3.14)	2.05 (1.22, 3.42) *
	Harari	1.46 (0.87, 2.44)	1.46 (0.84, 2.54)
	Dire Dawa	1.63 (0.97, 2.75)	1.70 (0.98, 2.94)
Mother’s education level	No education	1	1
	Primary	1.21 (1.04, 1.42)	1.05 (0.89, 1.25)
	Secondary/higher	0.99 (0.78, 1.25)	0.84 (0.64, 1.11)
Mother’s age in years	15–24	1	1
	25–34	0.89 (0.77, 1.05)	1.05 (0.88, 1.24)
	35–49	0.71 (0.58, 0.87)	0.86 (0.70, 1.06)
Residence	Urban	1	1
	Rural	1.17 (0.95, 1.13)	1.11 (0.83, 1.49)
Current age of children (month)	< 6	1	1
	6–11	2.66 (2.02, 3.52)	2.66 (2.01, 3.52) *
	12–23	2.53 (1.96, 3.26)	2.45 (1.89, 3.17) *
	24–35	1.61 (1.24, 2.10)	1.53 (1.17, 2.01) *
	36–47	1.05 (0.79, 1.38)	1.01 (0.76, 1.34)
	48–49	0.50 (0.37, 0.67)	0.49 (0.35, 0.66) *
Number of children under 5 years	1	1	1
	2	0.84 (0.72, 0.97)	0.86 (0.74, 1.01)
	≥ 3	0.68 (0.55, 0.82)	0.76 (0.61, 0.94) *
Mother’s occupation	Not working	1	1
	Working	1.18 (1.02, 1.35)	1.19 (1.03, 1.38) *
Wealth index	Poor	1	1
	Middle	1.15 (0.95, 1.41)	1.07 (0.87, 1.33)
	Rich	1.05 (0.89, 1.23)	1.08 (0.88, 1.33)
Source of drinking water	Improved water	1	1
	Unimproved water	1.04 (0.89, 1.21)	1.06 (0.90, 1.25)
Toilet facilities type	Improved water	1	1
	Unimproved water	1.15 (0.95, 1.39)	1.04 (0.82, 1.32)
Child stool disposal	Safe	1	1
	Unsafe	0.83 (0.71, 0.96)	1.07 (0.9, 1.26)
Media exposure	Exposed	1.04 (0.88, 1.23)	1.02 (0.85, 1.23)
	Not exposed	1	1

## Discussion

This study provided valuable insights into the factors affecting the prevalence of diarrhea. The study also highlighted the significant burden of diarrheal diseases across regions, with a particularly profound impact on the presence of spatial inequalities.

The national prevalence of childhood diarrhea (11.87%) was somewhat lower than that of a previous survey conducted in 2011 (15%) [24, 28] which might be due to differences in the study periods. There were significant variations in the prevalence of diarrhea in children between regions of residences, specifically in SNNP, Amhara, Oromia, Tigray, and Gambella regions [24, 31]. This disparity might be due to sample sizes, study period, latrine coverage, and utilization as well as access to safe water for drinking. However, this finding is consistent with those of other sub-Saharan and South Asian countries, for example, studies in Tanzania and India [20, 48–50]. The results signify that the strategic approach of the government is fruitful and has been focused on low-performing areas. These remarkable achievements are the results of various initiatives of the government. Since diarrhea in children is affected by multiple factors, the major contributing factors to diarrhea in children in different regions found in this study might be different. Despite this progress, we found that relative measures of spatial inequalities across the region show that the impact of space has become more pronounced among childhood illnesses in particular over the decades. This is suggesting that an upsetting trend should be closely monitored throughout the SDG era and further explored with new household surveys by including some important variables, such as cultural beliefs and perceptions.

In this study, having three or more under 5 years of age children was statistically significant for the occurrence of diarrheal disease in Ethiopia. As the number of children increased, the frequency of diarrhea decreased significantly. This finding is different from those previous studies done in northwest Ethiopia [31] and other sub-Saharan African countries [21, 48]. Ideally, it is expected that when the number of children in households increases, the vulnerability to contamination of children also increases because the quality of care and attention of parents decreases. However, our study suggests that national policy could potentially impact the decline of childhood illness at the level of the individual affecting day-to-day clinical practices. Recent measures, such as the Community Health Extension Program towards universal coverage of primary health services implemented in aggregation with such changes, may reduce adverse health effects [51, 52]. This is because multisectoral collaboration projects like those of UNICEF have been working on childhood education and family health to raise the awareness of mothers/caregivers on childcare. This suggests that Ethiopia has rapidly increased the number and scale of maternal health interventions across the country, such as family planning.

In this study, parental occupational status was more correlated to childhood diarrheal disease occurrence compared to parents with no occupation. This finding is in line with results in sub-Saharan countries [48] and northwest Ethiopia [53]. This could be because mothers/caregivers without work usually have sufficient time to control children to minimize their exposure. Moreover, caregivers/mothers have the opportunity to get information from different sources and to practice it. In contrast, working mothers are likely to have limited time to control their children as they need to spend much time on economic activities to increase family income than taking care of their children.

This study also noted a significant association between the age of the child and the occurrence of diarrheal disease. This finding is similar to other study findings conducted in Ethiopia and elsewhere [31, 48, 50]. Children aged 0–5 months have a low risk of diarrhea. During this time, all mothers are advised to give exclusive breastfeeding, which minimizes child exposure to contaminated agents since most children do not usually start complementary feeding before 6 months. However, children between the ages of 6 and 23 months experienced more risk of diarrhea compared to those 0–5 months. The possible reason for this could be that at this age children crawling and walking on the ground may have increased chances of exposure to pathogenic microorganisms. Moreover, since complementary feeding is common after 6 months, poor food handling and preparation might increase the chances of catching diarrheal diseases. The possible reason for the subsequent decrease in diarrhea after 23 months may be due to the development of immunity to pathogens after repeated exposures and immunization.

The findings of this analysis have important policy consequences for the design and implementation of the health system. The report indicated that participatory approaches to change the behavior of the populations were necessary to promote sanitation situations for communities. Generally, these results are particularly significant in terms of the creation of prevention programs against children’s diseases for the Ministry of Health, health insurance companies, and partners. Childhood diseases are a global problem that is occurring in high-, middle-, and low-income countries. However, it is more likely to occur in marginalized communities, often driven by poverty and lack of education and job opportunities. Through improving outreach programs and expanding the community service delivery network, coverage will be increased in all segments of the population. Structural factors that cause differential provision of health care resources by community-based primary care interventions using community health workers and volunteers who have, in the past, effectively contributed to narrowing the gap in inequality and increasing access need to be addressed. However, sub-national information on these inequalities may be required by quantifying the contributions attributable to each predictor and evaluating their changes over time. Prompt diagnosis and careful treatment of diarrhea are essential to reducing childhood morbidity and mortality. This study finds unusual geographic trends in childhood illness and highlights sub-national areas where the possible confluence of human, household, and environmental factors influencing children’s illness may be spatially clustered.

One of the strengths of this study is its use of nationally representative data that enable the findings to be generalized across the country. The other strength of this research is to have explored and quantified the extent of neighborhood differences in diarrheal morbidity in the regions. Therefore, the study findings can be used to inform policy and program managers. Some of the shortcomings of our study include sampling and measurement errors as well as recall and misclassification bias, self-reported issues, and lack of pathogen testing. Moreover, there is a warning that some regions used small samples, which could make the accuracy of the prevalence estimates per region questionable. In this report, the geographical coordinates of the enumeration areas (EAs) have been moved up to 5 km to avoid the identification of respondents or the population. This could have an impact on the cluster effect in the spatial analysis. So findings should be interpreted with caution.

## Conclusion

The average incidence of diarrhea over 2 weeks was typically clustered throughout Ethiopia. Also, this research found that two or more children under 5 years of age, maternal employment status, child age, and the region were significant factors in the incidence of diarrhea. This suggested that greater attention needs to be paid to reducing childhood diarrhea by focusing on maternal and child health across villages. In addition to the available strategies, customized interventions, regionally relevant resource provision, and awareness-raising programs will reduce the incidence of diarrhea and provide sufficient child health. Also, experience sharing and a better household climate may lead reduction in childhood illness.

## Data Availability

All relevant data are within the paper. However, data are available from the corresponding author upon reasonable request. Moreover, the datasets analyzed during the current study are available in the DHS repository, https://dhsprogram.com/data/available-datasets.cfm.

## Abbreviations

AIC: Akaike information criteria
AOR: Adjusted odds ratio
BIC: Bayesian information criteria
EAs: Enumeration areas
EDHS: Ethiopia Demographic and Health Survey
GLMM: Generalized linear mixed model
ICC: Intracluster correlation
LLR: Log-likelihood ratio
RR: Relative risk
SNNPR: South Nation Nationalities People Region
